# Editorial: Advances in the pathogenesis and treatment of osteoporosis: from bench to bedside

**DOI:** 10.3389/fmed.2025.1622559

**Published:** 2025-05-22

**Authors:** Wei Xie, Paolo Alberton, Mario Barbagallo, Eduardo Abreu, Dasheng Lin

**Affiliations:** ^1^Department of Orthopaedics and Trauma Surgery, Musculoskeletal University Center Munich (MUM), LMU University Hospital, LMU, Munich, Germany; ^2^Division of Hand, Plastic and Aesthetic Surgery, LMU University Hospital, LMU, Munich, Germany; ^3^Geriatric Unit, Department of Medicine, University of Palermo, Palermo, Italy; ^4^Muscle Biology Research Group, School of Nursing and Health Studies, University of Missouri–Kansas City, Kansas City, MO, United States; ^5^Center of Foot and Ankle Surgery, Beijing Tongren Hospital, Capital Medical University, Beijing, China; ^6^Department of Orthopedic Surgery, Fujian Medical University Union Hospital, Fuzhou, China

**Keywords:** osteoporosis, osteoporotic fracture, pathogenesis, treatment, risk factors

Osteoporosis is an age- and gender-associated musculoskeletal disorder characterized by compromised bone integrity. With the intensification of global population aging trends, the disease burden of osteoporosis is projected to escalate substantially. This demographic shift necessitates prioritized allocation of scientific and clinical resources toward two critical domains: enhancing fundamental understanding of disease mechanisms through multidisciplinary research and developing innovative therapeutic agents and intervention strategies targeting bone remodeling pathways. The articles in this topic covered the latest advancements in osteoporosis screening, etiology, risk factors, and treatment.

Currently, the relationship between Body Mass Index (BMI) and Bone Mineral Density (BMD) remains controversial. Some studies suggest that a higher BMI may promote bone formation by increasing body weight, thereby enhancing mechanical stimulation on bones (1). However, other research indicates that excessively high BMI may negatively affect bone metabolism due to obesity-related metabolic abnormalities (2, 3). The study by Chen et al. found that fat tissue distribution in different body regions shows significant correlations with BMD in corresponding bone areas. Excessively high regional fat percentages may be detrimental to bone health in both genders. Specifically, trunk fat percentage in females is significantly associated with lumbar spine BMD, while abdominal fat percentage in males shows strong correlations with femoral neck BMD. To promote bone health, males should limit waist circumference and avoid abdominal fat accumulation, whereas females should focus on controlling trunk circumference.

Obstructive sleep apnea syndrome (OSAS) is a sleep disorder characterized by intermittent hypoxia and sleep fragmentation, leading to oxidative stress and systemic inflammation (4). Luo X. et al. analyzed the correlation between OSAS and osteoarthritis (OA) using multivariate logistic regression analysis on 10,641 participants recruited from the National Health and Nutrition Examination Survey (NHANES) dataset. The results indicated that OSAS patients may have a higher prevalence of OA, and aging also plays a role in this association. After adjusting for covariates, it was found that aging mediates the relationship between OSAS and OA.

Patients with short bowel syndrome (SBS) experience insufficient intestinal absorption capacity due to partial resection of the small intestine (5). Long-term inappropriate parenteral or enteral nutrition can easily lead to complications such as metabolic bone disease (MBD) (Sun et al.). Sun et al. investigated the incidence and risk factors of osteopenia in adult SBS patients through a retrospective longitudinal cohort study. The study included 120 SBS patients, revealing that 76 patients (63.3%) developed osteopenia during the 10-year observation period. Given the high prevalence of metabolic bone disease among SBS patients, early identification and management of skeletal health issues in this population are crucial for preventing MBD.

Among the multifactorial causes of osteoporosis, the gut microbiota has become a focus of research due to its profound impact on bone metabolism (6). Luo Z. et al. conducted a bibliometric analysis of the literature on osteoporosis and the gut microbiota from 2014 to 2024 for the first time and explored the current research status, identifying the forefront and hotspots in this field. The study deeply explored fecal microbiota transplantation or specific dietary interventions as promising approaches for future research, providing references for researchers focused on this field.

Mendelian randomization (MR) is an epidemiological strategy that enhances causal inference by using single nucleotide polymorphisms (SNPs) as unbiased instrumental variables (IVs) (7). In the study by Dong et al., a two-way Mendelian randomization study was conducted to investigate the potential causal relationship between osteoporosis and 91 circulating inflammatory markers (CIMs). Based on a relatively large meta-analysis of genome-wide association studies (GWAS), they found a unidirectional positive causal relationship between CIMs and osteoporosis. Among them, five CIMs (ARTN, CXCL11, IL-18, LIF, and IFNG) showed potential associations with osteoporosis, possessing great value for further research.

Xiang et al. also applied MR methods to explore the causal relationship between Celiac's disease (CeD) and osteoporosis-related traits, while simultaneously examining the mediating role of inflammatory cytokines in the relationship between CeD and osteoporosis. It was found that the genetic susceptibility to CeD increases the risk of osteoporosis and osteoporotic fractures and reduces systemic BMD. Additionally, plasma IL-18 levels seem to play an important role in regulating the relationship between CeD and osteoporosis.

Long-term disuse osteoporosis (DOP) represents a critical health hazard for astronauts during extended spaceflight missions (8). While the regulatory role of long non-coding RNAs (lncRNAs) in bone marrow mesenchymal stem cells (BMSCs) and skeletal disorders has been established, the precise molecular mechanisms through which lncRNAs contribute to DOP pathogenesis remain poorly characterized. In a groundbreaking study, Wei et al. pioneered the construction of a competitive endogenous RNA (ceRNA) network in DOP-affected BMSCs, systematically integrating protein-coding mRNAs with non-coding RNA components (including lncRNAs and miRNAs). Through comprehensive analysis, the research team identified two pivotal hub genes (LAMC1 and LAMA4) and delineated two critical regulatory axes: the JPX/hsa-miR-3619-5p/LAMA4 cascade and the LINC01123/hsa-let-7i-5p/LAMC1 pathway. Notably, three pharmacological compounds—SB-216763, oxymetholone, and flubendazole—were computationally predicted as potential therapeutic agents targeting these molecular pathways.

Osteoporosis increases the risk of fragility fractures, especially of the lumbar and femoral fractures (9). Early detection of osteoporosis is crucial. Yoshida et al. created a dataset using bone mineral density and bilateral chest X-rays of 1,624 patients aged ≥20 years and proposed a new method using a deep learning model with anterior and lateral chest radiographs (CXR) inputs for early detection of osteoporosis. These results suggest that bidirectional CXR can improve the accuracy of BMD estimation and osteoporosis screening compared to single-view CXR and has the potential to serve as a criterion for clinical decision-making.

In the past few decades, percutaneous vertebroplasty (PVP) and percutaneous kyphoplasty (PKP) have been widely used to treat osteoporotic vertebral compression fractures (OVCF) due to their rapid pain relief and functional improvement effects (10). Understanding and effectively intervening with factors related to secondary fractures after discharge is crucial for improving patients' quality of life and avoiding future fractures. Yang et al. conducted a prospective analysis of OVCF patients who underwent PVP or PKP and further analyzed the risk factors for new vertebral fractures after treatment. It was found that for osteoporotic fracture patients undergoing PKP/PVP surgery, older age, poor blood glucose control, lower BMD, lower 25-OH-D3 levels, weaker paraspinal muscles, and higher fat infiltration were more prone to new vertebral fractures. On the other hand, maintaining regular physical activity and adhering to osteoporosis treatment can help prevent new vertebral fractures.

Osteoporosis and the fractures it causes remain a significant health challenge in the global aging society (11). However, the research compiled in this Research Topic systematically elucidates the “multi-factor, multi-target” pathogenesis of osteoporosis and precise intervention strategies by integrating multi-omics technologies, translational medical models, and clinical data. These groundbreaking discoveries not only promote the translation of basic research into clinical practice but also provide a new theoretical framework and practical guidance for developing individualized and staged precision prevention and treatment.
